# Photoassimilation, Assimilate Translocation and Plasmodesmal Biogenesis in the Source Leaves of *Arabidopsis thaliana* Grown Under an Increased Atmospheric CO_2_ Concentration

**DOI:** 10.1093/pcp/pcu004

**Published:** 2014-01-30

**Authors:** Zhongrui Duan, Ayumi Homma, Megumi Kobayashi, Noriko Nagata, Yasuko Kaneko, Yuki Fujiki, Ikuo Nishida

**Affiliations:** ^1^Laboratory of Plant Molecular Physiology, Division of Life Science, Graduate School of Science and Engineering, Saitama University, Shimo-Okubo 255, Sakura-Ku, Saitama, 338-8570 Japan; ^2^Department of Chemical and Biological Sciences, Faculty of Science, Japan Women’s University, 2-8-1 Mejirodai, Bunkyo-ku, Tokyo, 112-8681 Japan; ^3^Biology Section, Faculty of Education, Saitama University, Shimo-Okubo 255, Sakura-ku, Saitama, 338-8570 Japan; ^4^Institute for Environmental Science and Technology, Saitama University, Shimo-Okubo 255, Sakura-ku, Saitama, 338-8570 Japan

**Keywords:** *Arabidopsis thaliana*, CO2 atmospheric levels, Photoassimilate translocation, Plasmodesma formation

## Abstract

Using 18-day-old *Arabidopsis thaliana* seedlings grown under increased (780 p.p.m., experimental plants) or ambient (390 p.p.m., control plants) CO_2_ conditions, we evaluated ^14^CO_2_ photoassimilation in and translocation from representative source leaves. The total ^14^CO_2_ photoassimilation amounts increased in the third leaves of the experimental plants in comparison with that found for the third leaves of the control plants, but the rates were comparable for the first leaves of the two groups. In contrast, translocation of labeled assimilates doubled in the first leaves of the experimental group, whereas translocation was, at best, passively enhanced even though photoassimilation increased in their third leaves. The transcript levels of the companion cell-specific sucrose:H^+^ symporter gene *SUC2* were not significantly affected in the two groups of plants, whereas those of the sucrose effluxer gene *SWEET12* and the sieve element-targeted sucrose:H^+^ symporter gene *SUT4* were up-regulated in the experimental plants, suggesting up-regulation of SUT4-dependent apoplastic phloem loading. Compared with SUC2, SUT4 is a minor component that is expressed in companion cells but functions in sieve elements after transfer through plasmodesmata. The number of aniline blue-stained spots for plasmodesma-associated callose in the midrib wall increased in the first leaf of the experimental plants but was comparable in the third leaf between the experimental and control plants. These results suggest that *A. thaliana* responds to greater than normal concentrations of CO_2_ differentially in the first and third leaves in regards to photoassimilation, assimilate translocation and plasmodesmal biogenesis.

## Introduction

The atmospheric CO_2_ concentration has increased from 270 p.p.m. during pre-industrial times to 390 p.p.m. today and is predicted to be double the present concentration by the end of this century (IPCC 2007). An increase in the CO_2_ atmospheric concentration accelerates the photoassimilation rate in the source leaves of many plants, which may thereby enhance plant growth and consequently increase crop yield (Long et al. 2004, Long et al. 2006). For some herbs, however, increased atmospheric CO_2_ improves the photoassimilation rate over the short term but suppresses growth over the long term owing to suppression of photosynthetic gene expression induced by an overaccumulation of photoassimilates, i.e. sugars (Cheng et al. 1998). Thus, the proper balance between photosynthesis and translocation/use of photoassimilates is of crucial importance if plant growth is to be improved as the ambient atmospheric CO_2_ concentration increases worldwide.

Greater atmospheric CO_2_ concentrations have been shown to increase the concentrations of phytohormones, e.g. auxins, gibberellins and cytokinins, within apical zones (Teng et al. 2006) and may then promote plant cell division, elongation and differentiation (Yong et al. 2000, Li et al. 2002). Apical tissues, being sink tissues, require photoassimilates supplied by translocation from source tissues. Many studies have shown that plants grown under increased atmospheric CO_2_ concentrations have increased levels of soluble sugars and starch in their leaves, suggesting that photoassimilation increases as the CO_2_ concentration increases (Long and Drake 1992, Moore et al. 1997, Teng et al. 2006). These studies also suggested that the translocation and use of photoassimilates could be further optimized by increasing the relative atmospheric CO_2_ concentration. Grimmer and Komor (1999) reported that castor bean (*Ricinus communis* L.) plants grown under at 700 p.p.m. CO_2_ maintained a greater carbon export rate from leaves than did plants grown under ambient (350 p.p.m.) CO_2_ at night, whereas the export rates were comparable during the day, suggesting that the increased CO_2_ positively affected the castor bean translocation systems at night (Grimmer and Komor 1999). In general, however, the responses of translocation systems to increased CO_2_ have yet to be documented.

In most plants, sucrose, the primary photoassimilate in source leaves, is translocated to sink tissues via the phloem (Ruiz-Medrano et al. 2001). Sucrose produced in mesophyll cells of source leaves is symplastically transported to bundle sheath cells and phloem/vascular parenchyma cells of the minor veins via plasmodesmata. The major phloem-loading pathways diverge thereafter: in apoplastic phloem loading, sucrose is first exported to the apoplast by sucrose transporters and then taken up into the companion cell–sieve element (CC–SE) complex by sucrose:H^+^ symporters. During symplastic phloem loading, sucrose is loaded into specialized CCs, i.e. the intermediate cells, in which sucrose is converted into the oligosaccharides raffinose and stachyose via polymer trapping (Rennie and Turgeon 2009). In symplastic loaders, these oligosaccharides and sucrose are transferred into SEs via plasmodesmata (Rennie and Turgeon 2009). Different plant species use different phloem-loading pathways or different combinations of apoplastic and symplastic phloem-loading pathways because the distribution of plasmodesmata in the cell walls of their CC–SE complexes that contact different surrounding cells differs (Gamalei 1991). According to Gamalei’s morphological criteria, *Arabidopsis thaliana* is a Type 1–2a species, meaning that the species is an apoplastic phloem loader although a certain amount of raffinose is found in phloem exudates in addition to sucrose (Haritatos et al. 2000).

Apoplastic phloem-loading mechanisms have been most extensively studied in *A. thaliana*. Several crucial steps have been described at the molecular level, which involve the SWEET sucrose effluxers that engage in sucrose efflux from bundle sheath cells or phloem parenchyma cells into apoplasts (Chen et al. 2012) and involve the sucrose:H^+^ symporters SUC2 and SUT4 that control sucrose entry from the apoplast into CC–SE complexes (Sauer 2007). SUC2 is specifically expressed and functions in CCs (Schulze et al. 2003), whereas SUT4 is expressed in CCs (Schulze et al. 2003) but is targeted to the SE plasma membrane by transport of SUT4 protein from the CC to the SE through plasmodesmata (Kühn et al. 1997). Plants (*suc2* mutants) with a mutated *SUC2* are severe dwarfs and rarely fertile (Gottwald et al. 2000), demonstrating that SUC2 plays the central role in *A. thaliana* phloem loading, and SUT4 remains as the most plausible player in phloem loading in the *suc2* mutant.

Plasmodesmata are important for symplastic movement of sucrose. SEs are connected to neighboring CCs via plasmodesmata such that a functional CC–SE complex is formed, which is essential for sucrose phloem loading and translocation (Turgeon 1996). Plasmodesmata are ontogenically simple and linear in CC–SE walls, but branched or more complex forms develop in vein cell walls including those of CC–SE complexes during the sink to source transition. Plasmodesmata in CC–SE walls are important for sucrose entry into SEs and for the transfer of proteins that are functional in SEs, e.g. SUT4 and other cells after systemic translocation, e.g. FLOWERING LOCUS T (Yoo et al. 2013).

*Arabidopsis thaliana* has also been used as a model when studying responses to increased atmospheric CO_2_ concentrations (Lake et al. 2001, Ward and Kelly 2004, Teng et al. 2006, Ekman et al. 2007, Xin et al. 2009). These studies reported increased plant growth, increased carbohydrate and hormone concentrations, enhanced stomatal development, early flowering and altered lipid compositions associated with increased stroma to grana thylakoid ratios. However, the effects of increased atmospheric CO_2_ on photoassimilate translocation have not been studied to date. For this study, we investigated the effects of increased atmospheric CO_2_ on photoassimilation and translocation systems in *A. thaliana*. We show that the enhancement of photoassimilation and translocation found for plants grown under an increased atmospheric CO_2_ concentration is dependent on their source leaves and, with the use of reverse transcription–PCR (RT–PCR), we show that the increase in *SUT4* expression may be the cause of an increase in SUT4-dependent apoplastic phloem loading in these plants. Furthermore, aniline blue staining for plasmodesmal callose revealed the presence of an increased density of plasmodesmata in source leaf vein walls. We discuss the importance of plasmodesmal connections in the CC–SE walls in regard to sucrose and SUT4 entry into SEs.

## Results

### Growth of *A. thaliana* under increased atmospheric CO_2_ concentration

We compared the growth of experimental (780 p.p.m. CO_2_) and control (390 p.p.m. CO_2_) plants cultured under a 16 h light/8 h dark photoregime. In accordance with a previous study (Xin et al. 2009), the experimental plants developed leaves more rapidly (Supplementary Fig. S1A) and bolted earlier (Supplementary Fig. S1C) than did the control plants. On day 18, starches were strongly stained with iodine in the first to fourth leaves (source leaves) of the experimental plants (Supplementary Fig. S1B). Conversely, iodine-stained starch was almost absent in all leaves of the control plants (Supplementary Fig. S1B). These results confirmed that increased atmospheric CO_2_ promotes plant growth and photoassimilate accumulation in source leaves. On the other hand, under our growth conditions, the respiration capacity of detached rosette plants was not significantly affected by increased atmospheric CO_2_ concentration (Supplementary Fig. S2).

### Effects of increased atmospheric CO_2_ on photoassimilation and translocation

The phyllotaxis of *A. thaliana* follows a Fibonacci spiral, in which single organs are initiated successively at a divergence angle from the previous organ close to 137.5° (Refahi et al. 2010). After seed germination, *A. thaliana* develops the first and second leaves together and then the third and fourth leaves together; the fifth and higher leaves develop alternately later on (Supplementary Fig. 1A). Development of the first and second leaves must depend largely on photosynthetic competence in their corresponding cotyledons. In contrast, the third and fourth leaves must depend on photosynthetic competence in their corresponding cotyledons and the lower source leaves (first and second leaves). Accordingly, we anticipated that an increase in atmospheric CO_2_ might differentially affect the photoassimilation and translocation capacities of the source leaves and would depend on the leaf number. We therefore measured photoassimilation and translocation capacities of source leaves in the control and experimental plants using a ^14^CO_2_ pulse–chase protocol. The first or the third leaves of boltless 18-day-old seedlings, which were source leaves, were labeled with ^14^CO_2_ (∼598 p.p.m.) for 15 min. Then, the ^14^CO_2_ was substituted with air for 5 min, and labeled plants were inclubated for 40 or 100 min under ambient CO_2_ atmosphere. Next, the ^14^C label was visualized by radioimaging using a Bio-Image analyzer to determine the ^14^C-labeled photoassimilate distribution in different organs. Six plants grown under the same CO_2_ conditions were labeled with ^14^CO_2_ in the same chamber. Under such conditions, the six first leaf samples, grown at either 390 or 780 p.p.m., assimilated in total almost 0.18–0.19% of CO_2_ released in the acrylic chamber, whereas six third leaf samples, grown at 390 and 780 p.p.m., assimilated in total almost 0.34% and 0.82% of CO_2_ released in the acrylic chamber, respectively.

**Fig. 1:**
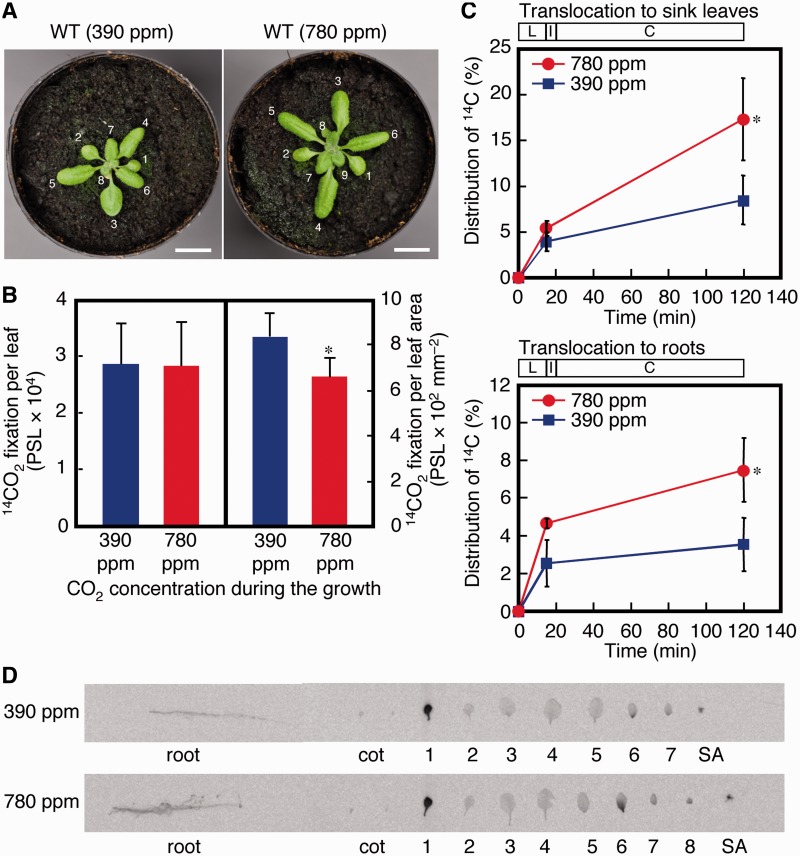
^14^CO_2_ assimilation in and translocation from the first leaf of 18-day-old *A. thaliana* plants grown under 390 p.p.m. (control) or 780 p.p.m. (experimental) CO_2_ concentration. (A) Photographs of typical wild-type (WT) 18-day-old *A. thaliana* plants used for the assimilation/translocation experiments. Left, a typical plant grown under a 390 p.p.m. (control) CO_2_ atmosphere. Right, a typical plant grown under a 780 p.p.m. (experimental) CO_2_ atmosphere. Each leaf is labeled with its order number. (B) ^14^CO_2_ photoassimilation by the first leaf. The plants had been grown under a 390 or 780 p.p.m. CO_2_ atmosphere for 18 d, after which their first leaves were exposed to ^14^CO_2_ for 15 min. Each result is the mean of three measurements with the associated error bar reported as the SD. *P < 0.05 (Student’s *t*-test). (C) Distribution of ^14^CO_2_ labels into sink leaves and roots. After 15 min exposure to ^14^CO_2_, labeled plants were transferred to an unlabeled CO_2_ atmosphere for 5 min and then incubated for up to 100 min. L, ^14^CO_2_ labeling for 15 min; I, 5 min interval; C, cold chase up to 100 min. Each result is reported as the mean ± SD for three replicates. *P < 0.05 (Student’s *t*-test). (D) Imaging of the radioactivity distributed among control and experimental plant tissues by a Bio-Imaging Analyzer FLA7000. Cot, cotyledons; SA, shoot apex.

For the first leaves of the experimental and control plants (Fig. 1A), their total ^14^CO_2_ photoassimilation amounts were not significantly different (Fig. 1B, left panel). However, the experimental plants had larger first leaves so that, when normalized to leaf area, the quantity of photoassimilates was 22% less for the experimental plants (Fig. 1B, right panel, *P* < 0.05). The experimental plants also exhibited a significantly greater extent of assimilate translocation to their sink leaves and roots than did the control plants (Fig. 1C, *P* < 0.05). After the 100 min chase, the control plants had exported 8.6% and 3.0% of the assimilated ^14^CO_2_ (15 min) to their sink leaves and roots, respectively, whereas for experimental plants the respective values were 17.4% and 6.3% (Supplementary Table S1). Therefore, the absolute amount of ^14^CO_2_ translocated to the sink leaves and roots of the experimental plants was approximately twice that of the control plants (Supplementary Table S1). The ^14^C distributions from the first leaves of the experimental and control plants to the higher ordered leaves showed that more photoassimilates were exported to the sixth leaves, which were just above the first leaves (i.e. sink leaves on the same orthostichy as the source) (Fig. 1D). The acropetal pattern of labeling, i.e. labeing in the base but not in the tip of the sixth leaves, suggested that they are sink to source transition leaves.

**Fig. 2:**
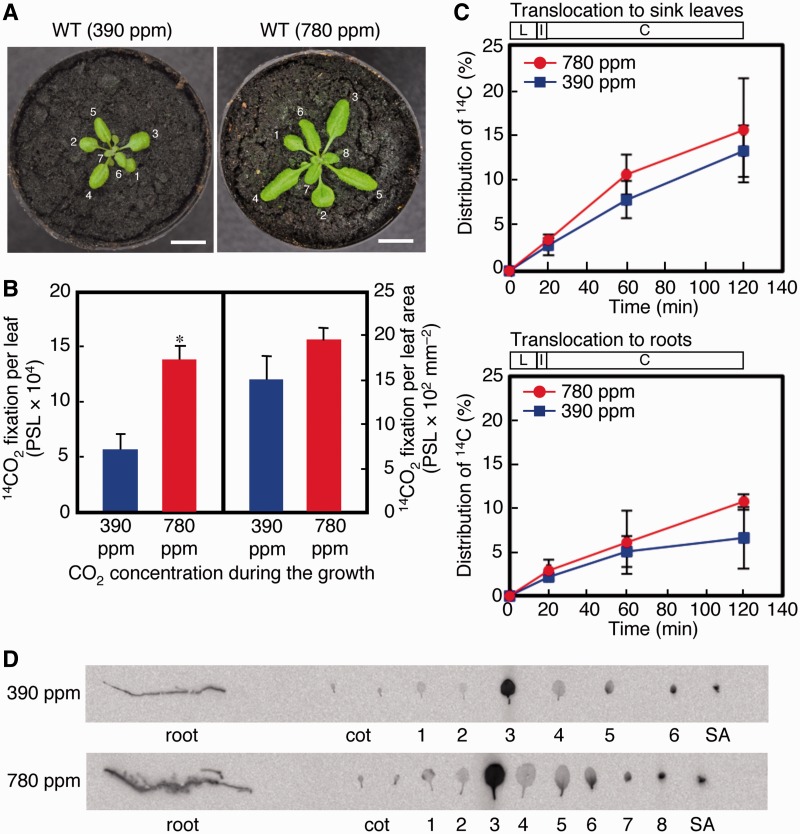
^14^CO_2_ assimilation in and translocation from the third leaf of 18-day-old *A. thaliana* plants grown under 390 p.p.m. (control) or 780 p.p.m. (experimental) CO_2_ concentration. (A) Photographs of typical wild-type (WT) 18-day-old *A. thaliana* plants used for the assimilation/translocation experiments. Left, control plant. Right, experimental plant. Each leaf is labeled with its order number. (B) ^14^CO_2_ assimilation in the third leaf. The pulse–chase experiment was performed as described in Fig. 1B. Each result is reported as the mean ± SD for three replicates. *P < 0.005 (Student’s *t*-test). (C) Distribution of ^14^CO_2_ in sink leaves and roots. Each result is the mean ± SD for three replicates. L, ^14^CO_2_ labeling for 15 min; I, 5 min interval; C, cold chase up to 40 min and 100 min. (D) Imaging of the radioactivity distributed among different tissues of the control and experimental plants by a Bio-Imaging Analyzer FLA7000. Cot, cotyledons; SA, shoot apex.

The effects of increased atmospheric CO_2_ were more noticeable for the total ^14^CO_2_ photoassimilation amounts of the third leaves. When the third leaves were labeled with ^14^CO_2_ (Fig. 2A), the experimental plants exhibited a significantly greater photoassimilation than did control plants (*P* < 0.005), i.e. a 2.4-fold greater amount per leaf and a 1.3-fold greater amount per leaf area (Fig. 2B; Supplementary Table S1). When normalized to sink leaves or roots, however, the distributions of ^14^C-labeled photoassimilates were comparable for the two groups (Fig. 2C). After the 100 min chase, the third leaves of the control and experimental plants had exported 13.3% and 15.6% of the assimilated ^14^CO_2_ to their sink leaves, respectively, and 5.6% and 10.7% to their roots, respectively (Supplementary Table S1). The absolute amounts of ^14^CO_2_ translocated to the sink leaves and roots were enhanced 2.9- and 4.0-fold, respectively, in the experimental plants (Supplementary Table S1), which suggests that translocation from the third leaf was substantially enhanced in the experimental plants. The ^14^C distributions in the different organs of both groups revealed that the third leaves exported photoassimilates mostly to leaves that were just above the source and had developed later than the fourth leaf (Fig. 2D) and that the sink to source transition of the sixth leaf occurred earlier in the experimental plants.

For control plants, the total amounts of ^14^CO_2_ photoassimilation of their third leaf were 1.8-fold greater per leaf and 1.7-fold greater per leaf area compared with the corresponding values for their first leaf. The differences in the total amounts of ^14^CO_2_ photoassimilation for the first and third leaves of the experimental plants were more pronounced: the third leaves exhibited a significantly greater photoassimilation (*P* < 0.001), i.e. 4.4-fold greater per leaf and 2.3-fold greater per leaf area.

In summary, the results demonstrated that photoassimilation and translocation are enhanced in the first and third leaves (acting as source leaves) of the experimental plants in comparison with those of control plants, i.e. a significantly enhanced translocation from the first leaves and a substantially enhanced translocation from the third leaves in parallel with a significantly enhanced accumulation of photoassimilates.

### Effects of increased atmospheric CO_2_ on expression of genes related to photosynthesis and translocation

**Fig. 3:**
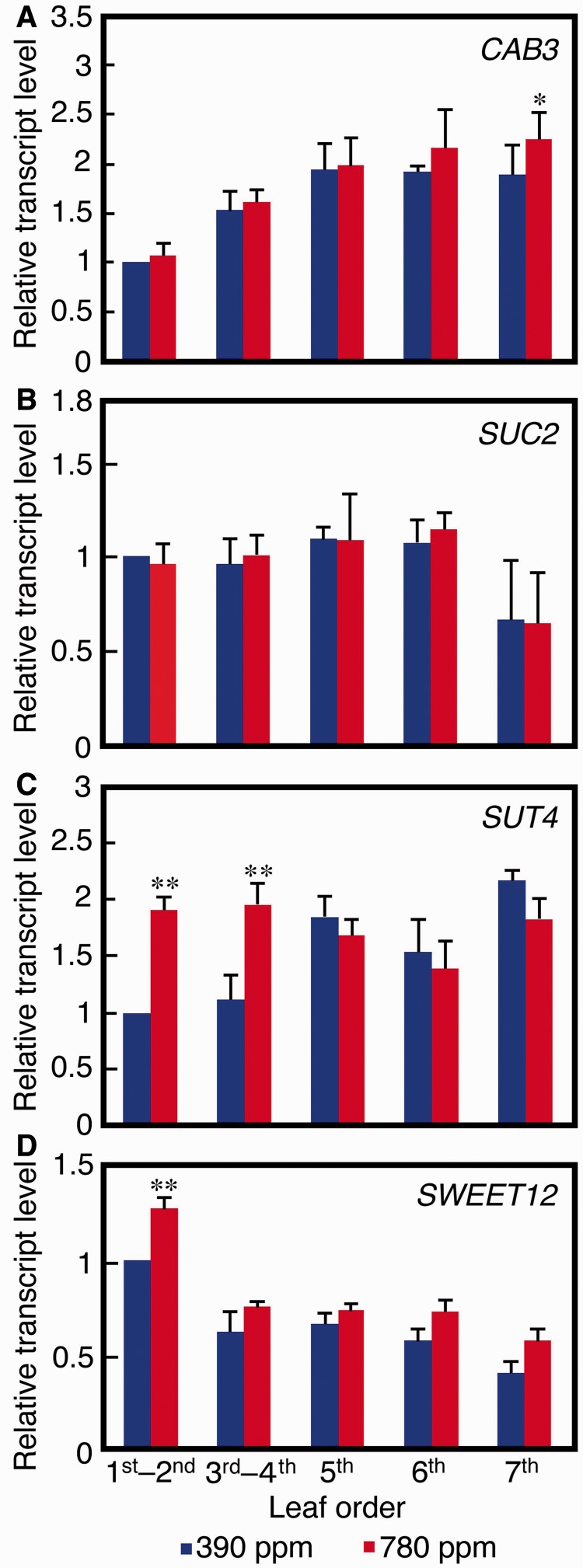
Relative transcription of genes related to photosynthesis and translocation in leaves of different order number from 18-day-old *A. thaliana* plants grown under a 390 p.p.m. (control) or a 780 p.p.m. (experimental) atmospheric CO_2_ concentration. Transcription levels of genes related to photosynthesis and translocation were measured by RT–PCR as described in the Materials and Methods. (A) Photosynthesis- and (B–D) translocation-related genes. The band intensities were quantified using ImageJ software. Each result is the mean ± SD (*n* = 3). *P < 0.01; **P < 0.001, for comparison of the control and experimental results for the given leaf orders using Tukey’s multiple comparison test.

To evaluate further the effects of increased atmospheric CO_2_ concentration on photosynthesis and translocation, we assessed the expression of genes related to photosynthesis and translocation with respect to the order of leaf appearance by RT–PCR, using samples harvested at 6 h of the light period (Fig. 3). The Chl *a/b* binding (CAB) protein is a component of the light-harvesting complex, hence it is required for photosynthesis (Mitra et al. 1989). *CAB3* expression in the first to sixth leaves of the experimental and control plants was comparable, whereas the seventh leaves (sink leaves) of the experimental plants showed significantly greater expression (Fig. 3A, *P* < 0.01).

SUC2 is a CC-specific sucrose:H^+^ symporter and the major regulator of apoplastic loading in *A. thaliana* (Gottwald et al. 2000). *SUC2* expression was approximately the same for all leaves in the control and experimental plants (Fig. 3B).

SUT4 is a sucrose:H^+^ symporter located in the SE plasma membrane and has been expected to play a minor role in phloem loading (Weise et al. 2000). In the experimental plants, however, the first and second leaves (1.9-fold, *P* < 0.001) and the third and fourth leaves (1.8-fold, *P* < 0.001) had significantly greater *SUT4* expression than did the corresponding control plant leaves. Conversely, *SUT4* expression was comparable for leaves that had developed later than the fourth leaves regardless of CO_2_ concentration (Fig. 3C).

SWEET12 is a major sucrose effluxer located in the plasma membrane of phloem parenchyma cells and catalyzes the first step of sucrose export to apoplasts during apoplastic phloem loading (Chen et al. 2012). *SWEET12* expression increased gradually with decreasing leaf order, and the first and second leaves of the experimental plants showed a significantly greater expression compared with the corresponding leaves of the control plants (Fig. 3D).

### Increased atmospheric CO_2_ increases aniline blue staining of plasmodesmal callose in the vein walls of source leaves

**Fig. 4:**
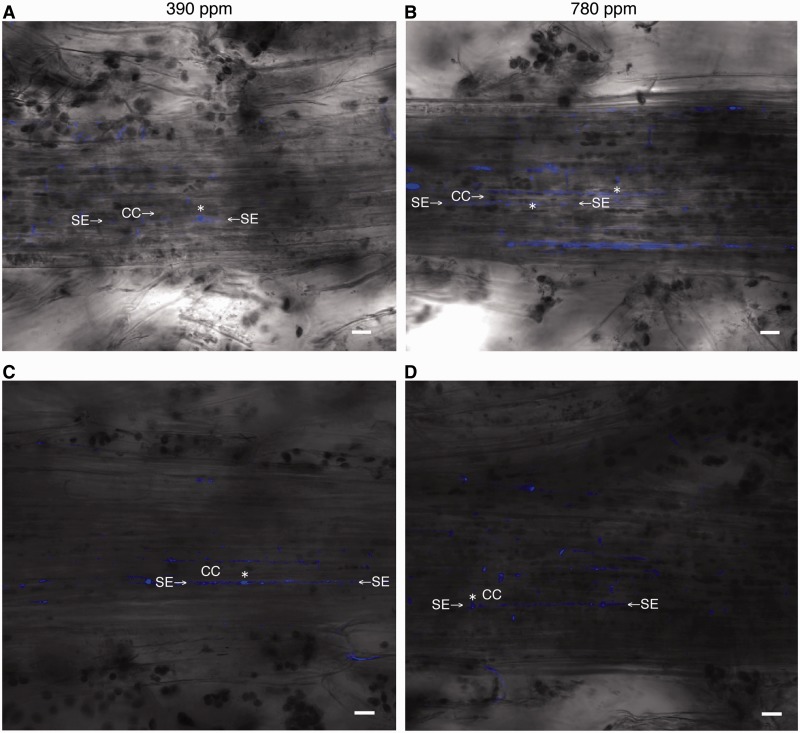
Aniline blue staining for plasmodesmal callose in the first and the third leaves of 18-day-old *A. thaliana* plants grown under a 390 p.p.m. (control) or a 780 p.p.m. (experimental) atmospheric CO_2_ concentration. Z-stacked confocal images of vein cells in the midrib of the first leaves (A, B) and the third leaves (C, D) of the control and experimental plants. (A) Control plants grown under a 390 p.p.m. CO_2_ atmosphere. (B) Experimental plants grown under a 780 p.p.m. CO_2_ atmosphere. Asterisks indicate sieve plates. SE, sieve element; CC, companion cell. Scale bars = 10 µm.

Callose is deposited in the necks of plasmodesmata in mature tissues (Bayer et al. 2004), and aniline blue staining for callose is used to identify plasmodesmata in conjunction with fluorescence microscopy (Simpson et al. 2009). In the first leaves of the experimental and the control plants, we detected aniline blue-stained plasmodesmal callose in their midribs along the SE walls at a density of 0.20 ± 0.05 spots µm^−1^ (*n* = 5) and 0.07 ± 0.02 spots µm^−1^ (*n* = 4), respectively (Fig. 4; Supplementary Table S2). These values were significantly different (*P* < 0.01), suggesting that plasmodesmal development was promoted in the first leaves of the experimental plants. In contrast, in the third leaves of the experimental and the control plants, we detected aniline blue-stained plasmodesmal callose in their midribs along the SE walls at a density of 0.20 ± 0.08 spots µm^−1^ and 0.19 ± 0.02 spots µm^−1^ (*n* = 4 for both groups), respectively (Fig. 4; Supplementary Table S2). These values were statistically not significant (*P* = 0.81), suggesting that plasmodesmal development occurred comparably in the third leaves of the experimental and the control plants.

### TEM analyses of plasmodesmal connections between CCs and SEs

**Fig. 5:**
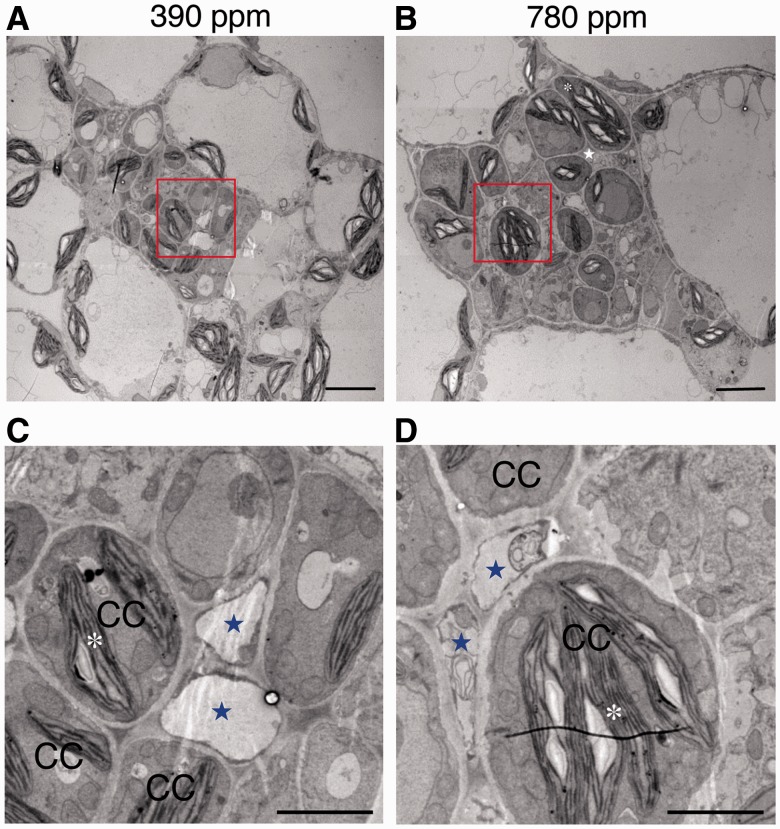
Electron micrographs in the minor veins of the first leaves of 18-day-old *A. thaliana* plants grown under a 390 p.p.m. (control) or a 780 p.p.m. (experimental) atmospheric CO_2_ concentration. Note that more and larger starch grains were observed in chloroplasts of vein cells (such as CCs) of the experimental plants (B) than the control plants (A). (C, D) Magnified images of the red boxes in (A) and (B), respectively. Blue stars indicate sieve elements. White asterisks indicate starch, CC, companion cell. Scale bars = 5 µm for (A) and (B); 2 µm for (C) and (D).

We used transmission electron microscopy (TEM) to observe cross-sections of the minor veins and midribs in the first leaves. In the minor vein sections from the experimental plants, we found relatively large starch grains in the chloroplasts of CCs and other vein cells. Conversely, the chloroplasts of the control first leaves in the corresponding cells displayed lesser or negligible amounts of starch grains (Fig. 5). These results supported our iodine staining experiments, which showed that the experimental plants contained large amounts of starch (Supplementary Fig. 1B). In the minor vein sections of the first leaves from control plants, each SE was connected to 2.5 ± 0.53 CCs [the average of two separate experiments with *n* = 5 (experiment 1) or *n* = 6 (experiment 2)], whereas in the minor vein sections of the first leaves from the experimental plants, each SE was connected to 2.9 ± 0.40 CCs [the mean of two separate experiments, with *n* = 6 (experiment 1) and *n* = 5 (experiment 2)]. These values are statistically the same for the two sets of plants. In the minor vein sections of the first leaves from control plants, the number of plasmodesmata was 0.67 ± 0.82 plasmodesmata per SE (experiment 2) or no plasmodesmata per SE (experiment 1), whereas in the minor vein sections of the first leaves from the experimental plants, the number of plasmodesmata was 1.2 ± 0.98 plasmodesmata per SE. These data are combined in Supplementary Table S3, which also indicates no significant difference. These values are again statistically the same for the two sets of plants. In the midrib of the first leaves, both experimental and control plants developed several types of complex plasmodesmata but they could not be defined as specific to either groups of the plants (Supplemental Fig. S3).

## Discussion

### Photoassimilation and translocation are enhanced in plants grown under increased atmospheric CO_2_ although different source leaves are affected differently

Using 18-day-old boltless Arabidopsis seedlings grown under a control (390 p.p.m.) CO_2_ atmosphere, we demonstrated that the first and third leaves are source leaves, that the sixth leaves are transitioning from sink to source conditions, and that leaves of orders greater than six are sink leaves (Figs. 1D, 2D). The number of leaves, the progression of the sink to source transition and the extent of leaf expansion are promoted by increased atmospheric CO_2_ concentration (Figs. 1D, 2D).

We showed that, although photoassimilation is promoted in the experimental plants compared with control plants, photoassimilation manifests differently in the first and third leaves. The total extent of photoassimilation of the first leaf of both types of plants was the same (Fig. 1B), although the extent per leaf area was less in the experimental plants compared with that of the control plants owing to an increase in leaf area for the experimental plants (Fig. 1B) rather than a suppression of photosynthetic gene expression (Fig. 3A). Conversely, the photoassimilation capacity was greater in the third leaves of experimental plants compared with control plants (Fig. 2B). These findings are consistent with our data showing that *CAB3* transcript levels are up-regulated in the seventh leaves (sink leaves) of the experimental plants. Therefore, an increase in atmospheric CO_2_ also seems to increase the photoassimilation capacity of the third and fourth leaves of *A. thaliana* and may be related to the observation that the development of the third and fourth leaves is promoted by an enhanced translocation rate of the first and second leaves in the experimental plants as compared with those of the control plants (discussed below).

We also showed that translocation is differentially promoted in the first and third leaves of the experimental plants as compared with those of control plants. The translocation capacity of the first leaves of the experimental plants was significantly up-regulated (almost doubled) compared with that of the first leaves of control plants (Fig. 1C). Conversely, the proportion of translocated ^14^CO_2_ to assimilated ^14^CO_2_ in the third leaves was the same in both CO_2_ conditions (Fig. 2C). In the experimental plants, however, the absolute amounts of ^14^CO_2_ translocated to the sink leaves and the roots were 2.6- and 3.6-fold greater than the corresponding values of the control plants.

We showed that source leaves translocated photoassimilates to sink leaves and the roots, which raises the issue of how the relative and different translocation rates to sink leaves and roots are maintained in different source leaves. In the control plants, the first and third leaves translocated ^14^CO_2_ assimilates into sink leaves and roots at ratios of 2.9 : 1 (74 : 26) and 2.0 : 1 (67 : 33), respectively. In the experimental plants, the first leaves translocated ^14^CO_2_ assimilates to sink leaves and roots in a similar proportion (ratio of 2.8 : 1; 74 : 26), whereas the third leaf had a translocation ratio of 1.5 : 1 (60 : 40) owing to a more pronounced translocation of photoassimilates to roots. Therefore, an increase in atmospheric CO_2_ concentration promoted translocation of photoassimilates from the third leaves to the roots more rapidly than to sink leaves, suggesting that the longer exposure to the increased CO_2_ concentration by the third leaves in comparison with the first leaves influenced the differences in the destinations of the photoassimilates from these two source leaves. We used the first and third leaves as representative source leaves to examine the export of photoassimilates to sink leaves and roots, knowing that the relative contributions of the first and third leaves in photoassimilate export had not previously been evaluated critically. We showed that the exported photoassimilates from the first and third leaves were found at a 1 : 2.8 ratio (26 : 74) for sink leaves and at a 1 : 4.0 (20 : 80) ratio for roots in the control plants and a 1 : 4.0 (20 : 80) ratio for sink leaves and a 1 : 7.6 (12 : 88) ratio for roots in the experimental plants. Thus, the third leaves relative to the first leaves are more important as a photoassimilate exporter for the experimental plants compared with the control plants.

### Increased atmospheric CO_2_ up-regulates *SUT4*-dependent apoplastic phloem loading

*Arabidopsis thaliana* is an apoplastic phloem loader, and SUC2-dependent phloem loading is its major phloem loading pathway. The recently characterized protein SWEET is involved in sucrose efflux to the apoplast. We found that *SWEET12* expression was up-regulated in the first and second leaves of the experimental plants, suggesting that up-regulation of apoplastic phloem loading in these leaves occurs when CO_2_ is at greater than ambient levels. Interestingly, however, the expression of *SUC2*, the major regulator of sucrose phloem loading in *A. thaliana*, was the same in all leaves of the experimental and control plants, whereas that of *SUT4*, a minor regulator, was significantly up-regulated in source leaves, i.e. the first, second, third and fourth leaves (Fig. 3C).

Given our findings, we propose a mechanism by which increased atmospheric CO_2_ promotes photoassimilation and translocation in 18-day-old *A. thaliana* seedlings. In the first and second leaves, an increase in atmospheric CO_2_ does not affect photoassimilation capacity but promotes translocation of photoassimilates by up-regulating SWEET12–SUT4-dependent apoplastic phloem loading. In the third and fourth leaves, an increase in CO_2_ concentration increases the photoassimilation capacity, which may be associated with up-regulation of photosynthetic genes such as *CAB3*. Translocation is passively enhanced in the third and fourth leaves as a result of increased photoassimilation, but may also be positively affected by the increased SUT4-dependent phloem loading.

### Increased atmospheric CO_2_ up-regulates plasmodesmal biogenesis

**Fig. 6:**
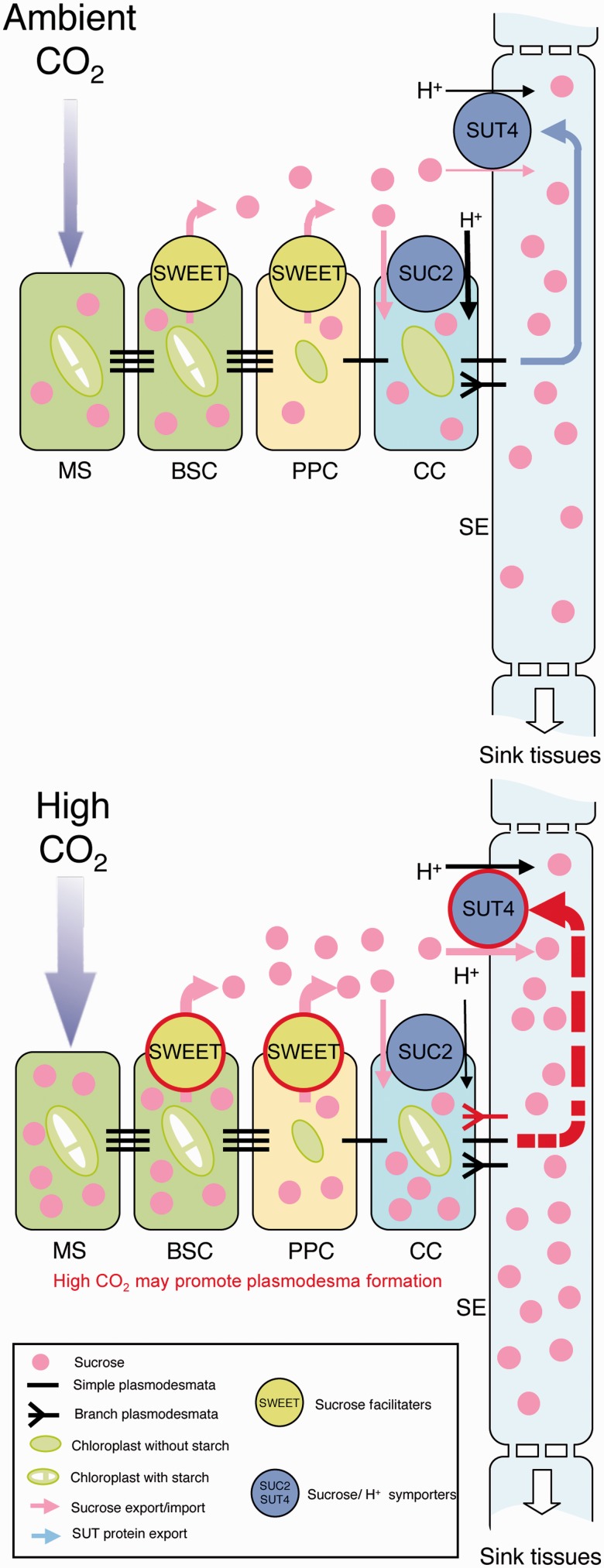
Model for the translocation from the first leaf of an 18-day-old *A. thaliana* plant grown under a 390 p.p.m. (control) or a 780 p.p.m. (experimental) atmospheric CO_2_ concentration. (A) Translocation of photoassimilates out of the first leaf of a plant grown under the control atmospheric CO_2_ concentration. Under a 390 p.p.m. atmospheric CO_2_ concentration, photoassimilated sucrose (pink circles) is loaded into the phloem (CC–SE complex) via the SUC2-dependent apoplastic pathway. (B) Translocation of photoassimilates out of the first leaf of a plant grown under the experimental atmospheric CO_2_ concentration. Under a 780 p.p.m. atmospheric CO_2_ concentration, the SWEET–SUT4-dependent apoplastic phloem loading pathway and plasmodesma biogenesis are up-regulated, and both facilitate up-regulation of translocation from the first leaf. The red circle lines of SWEET and SUT4 and red plasmodesma indicate up-regulation under a 780 p.p.m. atmospheric CO_2_ concentration, whereas a dashed red arrow line from CC to SUT4 indicates hypothesized up-regulation under the elevated CO_2_ concentration.

We also showed that plasmodesma formation appears to be enhanced in the midribs of the first leaves exposed to elevated atmospheric CO_2_, as we found an increased number of aniline blue-stained plasmodesmal callose spots in the midrib walls of the experimental plants when compared with those of control plants (Fig. 4). These results may be related to our finding that translocation is enhanced in the first leaves of experimental plants, possibly by assisting targeting of SUT4 to the plasma membrane of SEs and/or promoting efficient sucrose entry into SEs (Fig. 6). However, the molecular mechanisms of plasmodesmal formation along the sucrose translocation pathways remain to be characterized.

## Materials and Methods

### Plant materials and growth conditions

*Arabidopsis thaliana* (L.) Heynh (ecotype Columbia; Col-0) seeds were sown onto peat moss (Golden Peatban; Sakata Seed Corporation), irrigated with water and vernalized at 4°C for 2 d in the dark. Seedlings were grown at 23°C under a 16 h light/8 h dark photoregime with a photon flux density of 90–100 µmol m^−2 ^s^−1^ during the light period in CO_2_ pressure-controlled growth chambers (NC-220/350H; Nippon Medical & Chemical Instruments Co., Ltd.). One chamber was maintained at a 390 p.p.m. (ambient) CO_2_ concentration and the other at 780 p.p.m., using a CO_2_ controller (HC Type, Nippon Medical & Chemical Instruments). Plants were subjected to starch staining at 2 h of the light period and translocation experiments and RT–PCR sampling at 6 h of the light period.

### Starch staining

Whole rosettes from the plants were first decolorized by soaking them in 80% (v/v) ethanol at 90°C for 10 min. Next, the rosettes were stained with aqueous 39.4 mM I_2_/120 mM KI, after which they were washed twice with water.

### Photoassimilation and translocation

Entire plants except for a designated source leaf (the first or third leaf) were wrapped in aluminum foil and placed in an acrylic chamber (32 cm W × 30 cm D × 45 cm H) equipped with two electric fans for ^14^CO_2_ circulation. The acrylic chamber was placed in an open incubation chamber (140 cm W × 70 cm D × 130 cm H) regulated at 23°C and a photon flux density of 80 µmol m^−2 ^s^−2^ (CMP3244; OGAWA SEIKI Co., Ltd.). ^14^CO_2_ was then released inside the acrylic chamber by injecting 1 ml of propionic acid into 400 µl of 1 M NaH^14^CO_3_ (specific activity 0.09 GBq mmol^−1^; Moravek Biochemicals Inc.). The initial concentration of CO_2_ in the acrylic chamber was calculated as 598 p.p.m. After a 15 min exposure period, the chamber was flushed with air for 5 min, and the plants were then incubated for 40 or 100 min in the open incubation chamber. The leaves and roots of the plants were removed, and the distribution of radioactivity in these tissues was determined using a Bio-Imaging Analyzer (FLA-7000, Fujifilm). The radioactivity was expressed as photostimulated luminescence (PSL) values measured on FLA-7000. To quantify the radioactivity of the original NaH^14^CO_3_ solution, 10 µl of 20-fold diluted original solution (2.1 nmol NaH^14^CO_3_) was mixed with 10 µl of 10 mM NaOH and then blotted on filter paper for quantification using the Bio-Imaging Analyzer. The total amount of radioactive CO_2_ (3.7 MBq) released in the acrylic chamber was estimated to be 1.02 × 10^8^ PSL. Six plants grown under the same CO_2_ conditions were subjected to ^14^CO_2_ fumigation in the same chamber. Under such conditions, six first leaf samples in total, grown at 390 and 780 p.p.m., assimilated almost 0.19% and 0.18% of CO_2_ released in the acrylic chamber, respectively, whereas six third leaf samples in total, grown at 390 and 780 p.p.m., assimilated almost 0.34% and 0.82% of CO_2_ released in the acrylic chamber, respectively.

### RT–PCR

Leaves from the experimental and control plants were separated into their phyllotaxis (first and second leaves, third and fourth leaves, fifth leaf, sixth leaf, and seventh leaf), and then each group was frozen in liquid nitrogen. The frozen leaves were first pulverized, and total RNA was extracted from the frozen leaves using the RNeasy Plant Mini kit (Qiagen). cDNA was reverse transcribed from the total RNA using Transcriptor First Strand cDNA Synthesis kit reagents (Roche Applied Science), a PTC-100 thermal cycler (Bio-Rad), Ex-Taq polymerase (TAKARA BIO INC.) and gene-specific primers (Supplementary Table S4). The number of PCR cycles was 19 for *CAB3*, 26 for *SUC2*, 30 for *SUT4*, 28 for *SWEET12* and 25 for *Ubiquitin10*, with the temperature/time steps for a cycle as follows: 94°C for 30 s, 55°C for 30 s and 72°C for 80 s. The PCR program also includes an initiation step of 94°C for 5 min and a termination step of 72°C for 5 min. PCR bands were stained with 1 mg l^−1^ ethidium bromide, and the band intensities were quantified using ImageJ v. 1.46 (http://rsbweb.nih.gov/ij/) and normalized to the intensity of the *Ubiquitin10* band.

### Fluorescence of aniline blue-stained callose observed by confocal laser scanning microscopy

The first leaves from the plants were harvested, and the abaxial surface of their midribs was lightly scraped off by sliding a razor blade parallel to the abaxial leaf surface. The leaves were then stained with a mixture (2 : 3, v/v) of 0.1% (w/v) aniline blue (Wako) and 1 M glycerol, pH 9.5 (Simpson et al, 2009), and viewed 10 min later by confocal laser scanning microscopy (FV1000-D, OLYMPUS, using excitation and emission wavelengths of 405 nm and 460–500 nm, respectively. Fig. 4 shows a z-stacked image made from six optical section images, and some aniline blue-stained spots were overlapped together. Accordingly, the number of spots in Supplementary Table S2 was counted as the sum of single or double spots identified in any of the six optical section images.

### TEM

Leaves were fixed with 2% (v/v) glutaraldehyde/1% (w/v) paraformaldehyde in 50 mM sodium phosphate, pH 7.2, at 4°C overnight. The fixed samples were washed with 50 mM sodium phosphate, pH 7.2, fixed with 1% (w/v) osmium tetroxide in 50 mM sodium phosphate, pH 7.2 for 3 h at 4°C, dehydrated in acetone and embedded in Spurr’s resin (Polysciences, Inc.). Thin sections (70 m) were obtained using a diamond knife mounted on an ultramicrotome (ULTRACUT E, Leica), then stained with 4% (w/v) aqueous uranyl acetate for 18 min and with lead citrate solution for 7 min, and visualized by TEM (JEM-1400) at an accelerating voltage of 100 kV.

### Measurement of leaf respiration capacity

Leaf respiration capacity was measured according to Otsuru et al. (2013). Rosette leaves (50–80 mg FW) were sandwiched between nylon netting and inserted in a Clark-type oxygen electrode (6 ml chamber volume; Rank Brothers) containing 4 ml of assay buffer comprised of 50 mM HEPES (pH 6.6), 10 mM MES and 0.2 mM CaCl_2_. The TR capacity was defined as an O_2_ consumption rate in the dark in the absence of any inhibitor. COP capacity was defined as an O_2_ consumption rate in the dark in the presence of 15 mM SHAM (1 M stock in dimethylsulfoxide) that was sensitive to 2 mM KCN (1 M stock in assay buffer). AOP capacity was defined as an O_2_ consumption rate in the dark in the presence of 2 mM KCN that was sensitive to 15 mM SHAM.

## Statistical analysis

Statistical analyses were conducted using R version 2.12.1 (R Development Core Team 2010).

## Supplementary data

Supplementary data are available at PCP online.

## Funding

This work was supported by the Ministry of Education, Culture, Sports, Science, and Technology, Japan [Grant-in-Aid for Scientific Research on Innovative Areas (Nos. 22114503 and 24114703 to I.N.)].

## Disclosures

The authors have no conflicts of interest to declare.

## Supplementary Material

Supplementary Data

## Abbreviations

CC: companion cell
PSL: photostimulated luminescence
RT–PCR: reverse transcription–PCR
SE: sieve element
SUC: sucrose:H^+^ symporter
SUT: sucrose transporter
TEM: transmission electron microscopy
