# Supplementary material for: Photoassimilation, Assimilate Translocation and Plasmodesmal Biogenesis in the Source Leaves of Arabidopsis thaliana Grown Under an Increased Atmospheric CO2 Concentration
Source: Plant Cell Physiol. 2014 Jan 30;55(2):358–69. doi: 10.1093/pcp/pcu004 (PMC3913446; doi:10.1093/pcp/pcu004)
Supplement: Supplementary Data [file supp_55_2_358__index.html]

Photoassimilation, Assimilate Translocation, and Plasmodesmal Biogenesis In the Source Leaves of Arabidopsis thaliana Grown Under an Increased Atmospheric CO2 Concentration — Photoassimilation, Assimilate Translocation and Plasmodesmal Biogenesis in the Source Leaves of Arabidopsis thaliana Grown Under an Increased Atmospheric CO2 Concentration — Photoassimilation, Assimilate Translocation and Plasmodesmal Biogenesis in the Source Leaves of Arabidopsis thaliana Grown Under an Increased Atmospheric CO2 Concentration — Supplementary Data 

# Photoassimilation, Assimilate Translocation and Plasmodesmal Biogenesis in the Source Leaves of *Arabidopsis thaliana* Grown Under an Increased Atmospheric CO2 Concentration

## Supplementary Data

files

**Files in this Data Supplement:**

- Supplementary Data - pdf file
