# Supplementary material for: Photoassimilation, Assimilate Translocation and Plasmodesmal Biogenesis in the Source Leaves of Arabidopsis thaliana Grown Under an Increased Atmospheric CO2 Concentration
Source: Plant Cell Physiol. 2014 Jan 30;55(2):358–69. doi: 10.1093/pcp/pcu004 (PMC3913446; doi:10.1093/pcp/pcu004)
Supplement: Supplementary Data [file supp_pcu004_pcp-2013-e-00593-File008.pdf]

Supplementary Data

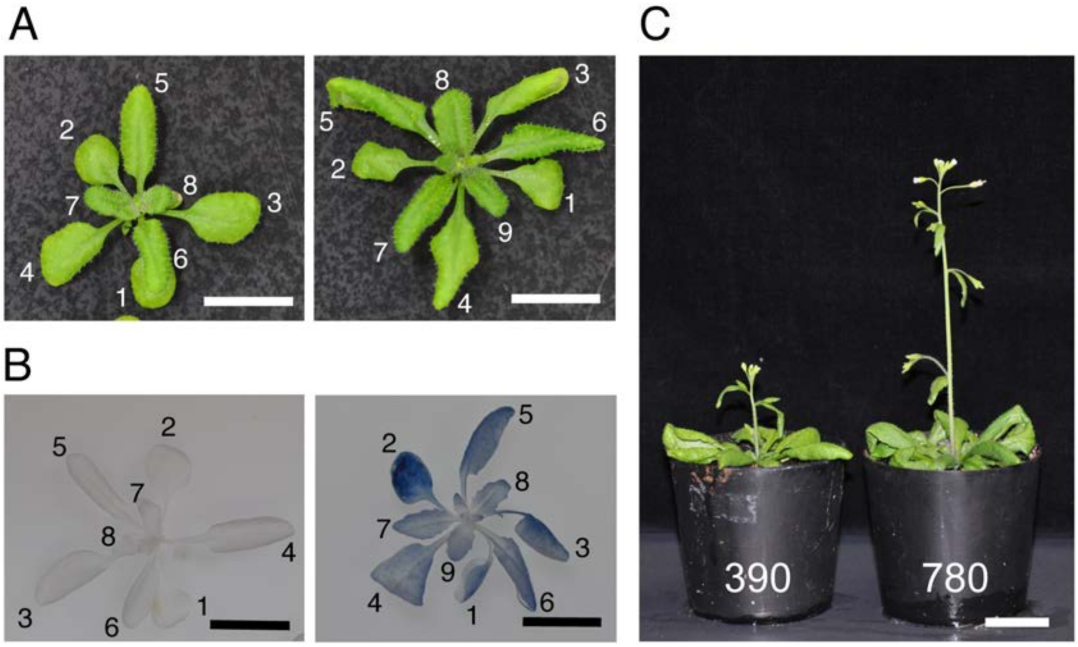

Supplementary Fig. S1 Photographs of *Arabidopsis thaliana* grown under ambient (390 ppm) or increased (780 ppm) atmospheric CO_2 concentrations.

(A) Plant grown for 18 days under a 390-ppm (left) or a 780-ppm (right) atmosphere of CO_2. (B) Leaves stained with iodine to visualize starch in a plant grown for 18 days under a 390-ppm (left) or a 780-ppm (right) CO_2 atmosphere. (C) Plants grown for 4 weeks under a 390-ppm (left) or a 780-ppm (right) CO_2 atmosphere of CO_2. Bars = 1 cm.

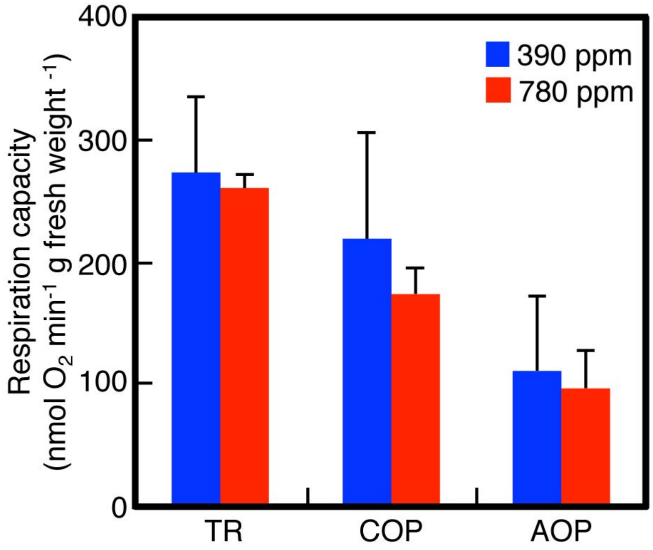

Supplementary Fig. S2 Respiration capacity in the rosette leaves of 18-d-old *A. thaliana* grown under a 390-ppm (control) or 780-ppm (experimental) atmospheric CO_2 concentration. The TR capacity was measured at 25°C in the absence of any inhibitor, whereas COP and AOP respiration capacities were measured in the presence of 15 mM SHAM and 2 mM KCN, respectively. Bars indicate the mean ± SD (n = 4). AOP, alternative oxidase pathway; COP, cytochrome oxidase pathway; TR, total respiration.

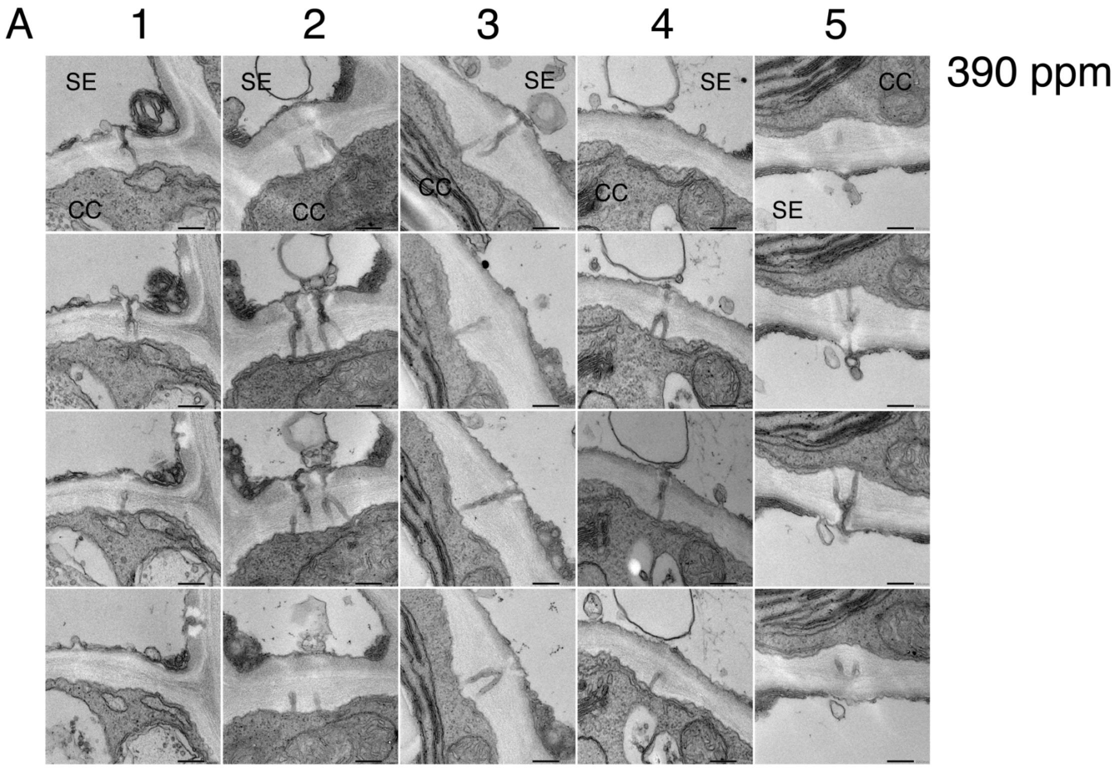

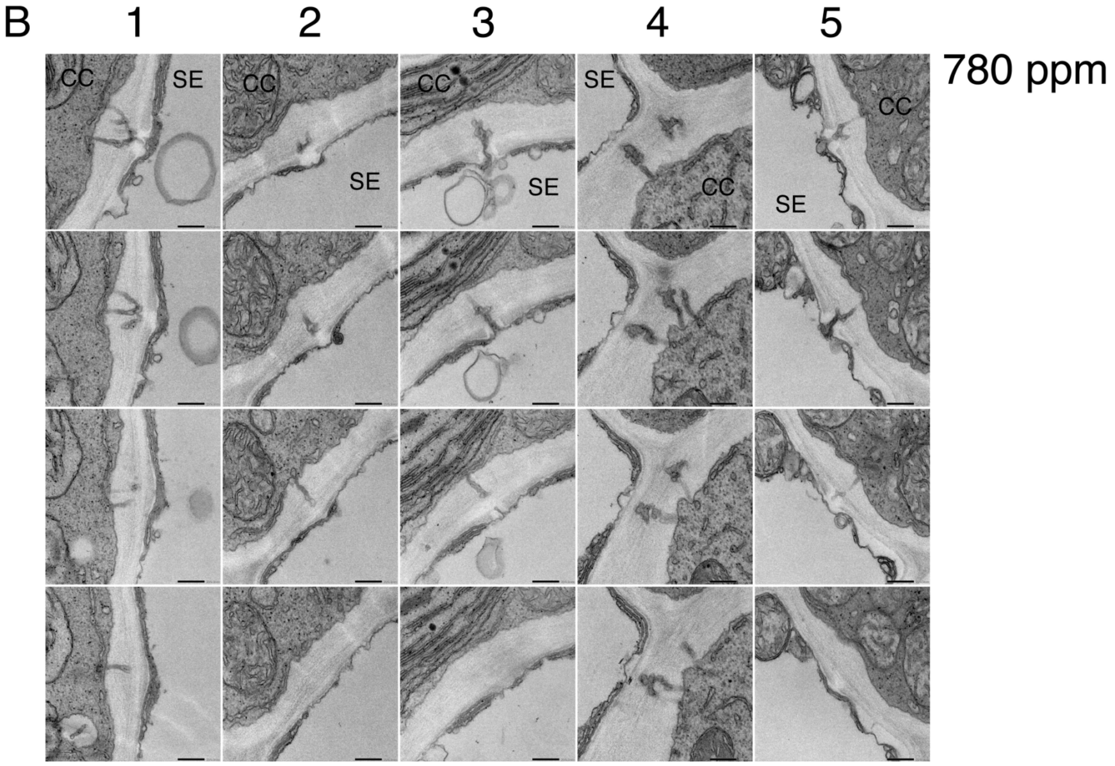

Supplementary Fig. S3 The ultrastructure of plasmodesmata in the midrib SE walls of the first

leaf of an 18-d-old Arabidopsis plant grown under a 390-ppm (A, control) or a 780-ppm (B, experimental) atmospheric CO_2 concentration. Numbers above the panels indicate different positions of plasmodeamata, and four serial sections are displayed for each position. SE, sieve element; CC, companion cell. Bars = 200 nm.

Supplementary Table S1. Photoassimilation and translocation capacities for the first and third leaves of 18-d-old *Arabidopsis thaliana* plants grown under a 390-ppm or a 780-ppm atmospheric CO_2 concentration

	$^{14}CO_2$ assimilation		Translocation	
	Per leaf	Per mm^2 leaf area	To sink leaves	To roots
CO_2 (ppm)	PSL[a] (fold)[b] (%)[c]	PSL[a] (fold)[b]	PSL[a] (fold)[b] (%)[c]	PSL[a] (fold)[b] (%)[c]
First leaf				
390	31,789 ± 8,623 (1.0) (100)	867 ± 68 (1.0)	2,728 ± 742 (1.0) (8.6)	949 ± 259 (1.0) (3.0)
780	31,434 ± 8,249 (0.99) (100)	680 ± 82 (0.78)	5,473 ± 1,435 (2.0) (17.4)	1,947 ± 520 (2.1) (6.3)
Third leaf				
390	57,518 ± 13,902 (1.0) (100)	1,501 ± 280 (1.7)	7,650 ± 1,849 (1.0) (13.3)	3,796 ± 918 (1.0) (6.6)
780	140,219 ± 11,865 (2.4) (100)	1,964 ± 159 (2.3)	21,874 ± 1,851 (2.9) (15.6)	15,003 ± 1,270 (4.0) (10.7)

[a]PSL, photostimulated luminescence.

[b]A fold activation relative to 390 ppm

[c]A percentage of ^{14}C-photoassimilated labels distributed to different tissues.

Supplementary Table S2. Total number of aniline blue-stained plasmodesmal callose spots in the midrib of the first and third leaves of 18-d-old *Arabidopsis thaliana* plants

Number of aniline blue-stained spots in the first leaves (390 ppm)[a]	Plasmodesma density (spot mm^{-1})	Number of aniline blue-stained spots in the first leaves (780 ppm)[a]	Plasmodesma density (spot mm^{-1})
11	0.05	32	0.16
12	0.06	36	0.18
18	0.09	30	0.15
12	0.06	51	0.25
		52	0.26
Average ± SE	0.07 ± 0.02	Average ± SE	0.20 ± 0.05 ($P = 0.0018$)

Number of aniline blue-stained spots in the third leaves (390 ppm)[b]	Plasmodesma density (spot mm^{-1})	Number of aniline blue-stained spots in the third leaves (780 ppm)[b]	Plasmodesma density (spot mm^{-1})
43	0.22	28	0.14
37	0.19	25	0.13
32	0.16	56	0.29
39	0.20	50	0.26
Average ± SE	0.19 ± 0.02	Average ± SE	0.20 ± 0.08 ($P = 0.8132$)

[a]The spots were counted along a SE wall length of 202.5 μm.

[b]The spots were counted along a SE wall length of 195.3 μm.

Supplementary Table S3. Statistical analysis of the occurrence of plasmodesmata in the cell walls connecting sieve elements (SEs) and companion cells (CCs) in the first leaves (minor vein section)[a]

	Count of SE cells having different numbers of plasmodesmata (observed)				
Plasmodesma number	0	1	2	3	Total
390 ppm CO_2	8	2	1	0	11
780 ppm CO_2	3	4	3	1	11
Total	11	6	4	1	22
					(χ^2 = 4.94)[b]

[a]χ^2 tests were conducted to test a null hypothesis that a proportion of each of SE cells having different numbers of plasmodesmata across CCs is not different between plants grown under 390 and 780 ppm CO_2. Data were obtained from two independent experiments.

[b]The χ^2 value is small enough ($\chi^2 < 7.81$) to mistakenly reject the null hypothesis ($P < 0.05$).

Supplementary Table S4. Primer sequences

Primer	Sequence
CAB3-Fw	5'-AGTTTCAATGGCCGCCTCCACA-3'
CAB3-Rv	5'-TAGCAAGACCCAATGGGTCGAAG-3'
SUC2-Fw	5'-GGTGTTGAATGGATTGGTCGGA-3'
SUC2-Rv	5'-AAGAGAGCCAAACAACCACTGC-3'
SWEET12-Fw	5'-CGTCACCATCAACAGCTTTG-3'
SWEET12-Rv	5'-AGTAGTTGCAGCACTGTTTCTA-3'
SUT4-Fw	5'-AAGCGAGAAATGGCTACTTCC-3'
SUT4-Rv	5'-TAGCCATTTGCAACTCGGGTT-3'
UBQ10-F	5'-GCCAAGATCCAAGACAAAGA-3'
UBQ10-R	5'-TTACGAGCAAGCATCATCAA-3'
